# Supplementary material for: Concurrent training in cardiac rehabilitation: A scoping review of aerobic-strength combinations in patients with coronary artery disease
Source: PLoS One. 2026 Mar 6;21(3):e0344208. doi: 10.1371/journal.pone.0344208 (PMC12965582; doi:10.1371/journal.pone.0344208)
Supplement: S1 File — The table presents the complete search strings, limits, filters, and retrieval dates applied in PubMed (MEDLINE), Scopus, Web of Science Core Collection, and the Cochrane Library (CENTRAL). The search strategy incorporated terms related to aerobic training, resistance training, cardiac rehabilitation, and coronary artery disease. Limits were applied according to each database’s functions, including study type, English language, adult populations, and publication years (2000–2025), with preprints excluded when applicable. The final number of records retrieved from each database is provided. (PDF) [file pone.0344208.s001.pdf]

Full Electronic Search Strategy (October 2023; updated Dec 2025)

Database	Search Date	Search Terms	Limits/Filters	Records Retrieved
**PubMed (MEDLINE)**	20 Oct 2023; updated 2nd Dec 2025	(((((("resistance training"[Title/Abstract]) OR ("strength training"[Title/Abstract])) OR ("weight training"[Title/Abstract])) OR ("resistance exercise"[Title/Abstract])) OR ("muscle training"[Title/Abstract])) AND ((("cardiac rehabilitation") OR ("exercise therapy")) OR ("rehabilitation"))) AND ((((("coronary artery disease"[Title/Abstract]) OR ("ischemic heart disease"[Title/Abstract])) OR ("myocardial infarction"[Title/Abstract])) OR ("coronary heart disease"[Title/Abstract])) OR ("CAD"[Title/Abstract]))	Full text, Clinical Conference, Clinical Study, Clinical Trial, Observational Study, Randomized Controlled Trial, English, Humans, Adult: 19+ years, Exclude preprints. Year range: 2000-2025	49
**Scopus**	22 Oct 2023; updated 3rd Dec 2025	((TITLE-ABS-KEY ("aerobic training") OR TITLE-ABS-KEY ("endurance training") OR TITLE-ABS-KEY ("cardiorespiratory fitness") OR TITLE-ABS-KEY ("aerobic exercise"))) AND ((TITLE-ABS-KEY ("resistance training") OR TITLE-ABS-KEY ("strength training") OR TITLE-ABS-KEY ("weight training") OR TITLE-ABS-KEY ("resistance exercise") OR TITLE-ABS-KEY ("muscle training"))) AND ((TITLE-ABS-KEY ("cardiac rehabilitation") OR TITLE-ABS-KEY ("exercise therapy") OR TITLE-ABS-KEY (rehabilitation))) AND ((TITLE-ABS-KEY ("coronary artery disease") OR TITLE-ABS-KEY ("ischemic heart disease") OR TITLE-ABS-KEY ("myocardial infarction") OR TITLE-ABS-KEY ("coronary heart disease") OR TITLE-ABS-KEY (CAD)))	English; All open access Year range: 2000-2025	164

**Web of Science (Core Collection)**	23 Oct 2023; updated 3rd Dec 2025	TS=("aerobic training" OR "endurance training" OR "aerobic exercise" OR "cardiorespiratory fitness") AND TS=("resistance training" OR "strength training" OR "weight training" OR "resistance exercise" OR "muscle training") AND TS=("cardiac rehabilitation" OR "exercise therapy" OR rehabilitation) AND TS=("coronary artery disease" OR "ischemic heart disease" OR "myocardial infarction" OR "coronary heart disease" OR CAD)	English; Open access<br>Year range: 2000-2025	75
**Cochrane Library (CENTRAL)**	23 Oct 2023; updated 3rd Dec 2025	("aerobic training":ti,ab,kw OR "endurance training":ti,ab,kw OR "aerobic exercise":ti,ab,kw OR "cardiorespiratory fitness":ti,ab,kw) AND ("resistance training":ti,ab,kw OR "strength training":ti,ab,kw OR "weight training":ti,ab,kw OR "resistance exercise":ti,ab,kw OR "muscle training":ti,ab,kw) AND ("cardiac rehabilitation":ti,ab,kw OR "exercise therapy":ti,ab,kw OR rehabilitation:ti,ab,kw) AND ("coronary artery disease":ti,ab,kw OR "ischemic heart disease":ti,ab,kw OR "myocardial infarction":ti,ab,kw OR "coronary heart disease":ti,ab,kw OR CAD:ti,ab,kw)	English<br>Year range: 2000-2025	135
