# Supplementary material for: Concurrent training in cardiac rehabilitation: A scoping review of aerobic-strength combinations in patients with coronary artery disease
Source: PLoS One. 2026 Mar 6;21(3):e0344208. doi: 10.1371/journal.pone.0344208 (PMC12965582; doi:10.1371/journal.pone.0344208)
Supplement: Attachment — Submitted filename: Response to Reviewers.docx [file pone.0344208.s004.docx]

**RESPONSE TO EDITOR**

| EDITOR’S LETTER | **Comments** | **Rebuttal** | **Location (line numbers)** |
| --- | --- | --- | --- |
|  | PONE-D-25-52063  Concurrent training in cardiac rehabilitation: a scoping review of aerobic-strength combinations in patients with coronary artery disease  PLOS ONE  Dear Dr. Latir,  Thank you for submitting your manuscript to PLOS ONE. After careful consideration, we feel that **it has merit but does not fully meet PLOS ONE’s publication criteria as it currently stands**. Therefore, we invite you to submit a revised version of the manuscript that addresses the points raised during the review process.  Please submit your revised manuscript by **Dec 20 2025 11:59PM**. If you will need more time than this to complete your revisions, please reply to this message or contact the journal office at [plosone@plos.org](mailto:plosone@plos.org). When you're ready to submit your revision, log on to https://www.editorialmanager.com/pone/ and select the 'Submissions Needing Revision' folder to locate your manuscript file.  Please include the following items when submitting your revised manuscript:   - **A rebuttal letter that responds to each point raised by the academic editor and reviewer(s).** You should upload this letter as a separate file labeled 'Response to Reviewers'. - **A marked-up copy of your manuscript that highlights changes made to the original version.** You should upload this as a separate file labeled 'Revised Manuscript with Track Changes'. - **An unmarked version of your revised paper without tracked changes.** You should upload this as a separate file labeled 'Manuscript'.   **If you would like to make changes to your financial disclosure, please include your updated statement in your cover letter.** Guidelines for resubmitting your figure files are available below the reviewer comments at the end of this letter.  If applicable, we recommend that you deposit your laboratory protocols in protocols.io to enhance the reproducibility of your results. Protocols.io assigns your protocol its own identifier (DOI) so that it can be cited independently in the future. For instructions see: <https://journals.plos.org/plosone/s/submission-guidelines#loc-laboratory-protocols>. Additionally, PLOS ONE offers an option for publishing peer-reviewed Lab Protocol articles, which describe protocols hosted on protocols.io. Read more information on sharing protocols at <https://plos.org/protocols?utm_medium=editorial-email&utm_source=authorletters&utm_campaign=protocols>.  We look forward to receiving your revised manuscript.  Kind regards,  Juan M. Murias Academic Editor PLOS ONE  Journal Requirements:  When submitting your revision, we need you to address these additional requirements. | Dear Editorial Team,  Thank you for your guidance regarding the resubmission requirements.  We confirm that all requested materials have been uploaded accordingly:   - A detailed **rebuttal letter** responding to each comment from the Academic Editor and reviewers has been submitted as a separate file titled **“Response to Reviewers”**. - A **marked-up version** of the revised manuscript with tracked changes has been uploaded as **“Revised Manuscript with Track Changes”**. - A **clean, unmarked version** of the revised manuscript has been uploaded as **“Manuscript”**.   In addition, the **updated financial disclosure statement** has been included in the **cover letter**, as requested.  Thank you for your consideration. Please let us know if any further information or revisions are required. | NA |
|  | 1. **Please ensure that your manuscript meets PLOS ONE's style requirements**, including those for file naming. The PLOS ONE style templates can be found at  https://journals.plos.org/plosone/s/file?id=wjVg/PLOSOne_formatting_sample_main_body.pdf and  https://journals.plos.org/plosone/s/file?id=ba62/PLOSOne_formatting_sample_title_authors_affiliations.pdf | We have reviewed and revised the manuscript to ensure full compliance with PLOS ONE’s style requirements, including formatting of the title page, author affiliations, main text, and file naming, in accordance with the official PLOS ONE templates. | All documents submitted |
|  | 2. We note you have included a table to which you do not refer in the text of your manuscript. **Please ensure that you refer to Table 3 in your text**; if accepted, production will need this reference to link the reader to the Table. | Thank you for the clarification. We have now ensured that **Table 3 is explicitly referred to within the Results section** of the manuscript. The updated version includes a clear in-text citation so that readers are properly directed to the table. | Line 247-249 |
|  | 3. **If the reviewer comments include a recommendation to cite specific previously published works, please review and evaluate these publications to determine whether they are relevant and should be cited.** There is no requirement to cite these works unless the editor has indicated otherwise. | We carefully reviewed the reviewer comments and confirm that no specific previously published works were recommended for citation. As such, no additional citations were added in response to this comment. The reference list remains focused on studies directly relevant to the scope, objectives, and inclusion criteria of the present review. |  |
|  | **Additional Editor Comments:**  In general, **both reviewers are supportive of the work** that has been presented, but some **methodological issues that need attention have been highlighted**. |  |  |

**RESPONSE TO REVIEWER 1**

| Reviewer #1: Summary | **Comments** | **Rebuttal** | **Location (line numbers)** |
| --- | --- | --- | --- |
|  | This manuscript examines the role of concurrent training (CT)—the integration of aerobic and strength exercise—within cardiac rehabilitation (CR) for patients with coronary artery disease (CAD). The authors describe the study as a scoping review conducted according to PRISMA-ScR guidelines and registered on the Open Science Framework. Fourteen clinical trials were included.  The review concludes that CT improves aerobic capacity, muscle strength, and functional performance, while effects on physical activity (PA) and health-related quality of life (HrQoL) remain inconsistent.  The topic is clinically important, timely, and relevant to advancing exercise-based CR. However, the manuscript shows a conceptual mismatch between its stated design (scoping review) and the methodology and interpretation used (which align more closely with a systematic review). Substantial revision is required to achieve conceptual and methodological coherence. |  | NA |
|  | **Major Comments** Introduction  In the final paragraph of the introduction, the authors state that “a systematic synthesis of available evidence is warranted” but then identify the study as a scoping review. This creates **terminological inconsistency** from the outset.  If the **intent was to “characterise and map” CT protocols**, the term “scoping review” is appropriate, but the outcome synthesis should remain descriptive.  If the goal is to evaluate the effectiveness of CT, the study should be reframed and registered as a systematic review. | We thank the reviewer for pointing out this inconsistency. Our intention is **not** to evaluate the effectiveness of concurrent training but to **map and describe** how CT protocols have been structured and implemented within cardiac rehabilitation.  We have therefore revised the final paragraph of the Introduction to accurately reflect the purpose and scope of the review. The revised text now clearly states that this study is a **scoping review designed to characterise CT protocols**, consistent with PRISMA-ScR guidance. No evaluative or effectiveness-based claims are made. | Line 94-98 |
|  | Methods  Several methodological issues warrant attention:  The **restriction to clinical trials only contradicts the exploratory scope of a scoping review,** which typically includes a broader range of study designs. **Revise the inclusion criteria to justify why only clinical trials were selected** or **broaden eligibility to include other study designs** consistent with scoping review methodology.  The **search strategy is not fully presented**, and there is **no mention of grey literature, conference abstracts, or manual reference checking**. | We thank the reviewer for this constructive feedback. In response, we have comprehensively revised the Methods section to align fully with scoping review methodology. All changes have been incorporated into the manuscript as detailed below.  We agree that limiting inclusion to clinical trials was inconsistent with the purpose of a scoping review. Therefore, we have expanded the eligibility criteria to include a wider range of empirical study designs.  We have revised the Search Strategy subsection to:   - describe all additional sources searched - add full search strategies for each database in Supplementary File 1 - clarify filters and limits used - explain the updated search (2023 → 2025) and how this affected the number of retrieved articles   **New additions now included** clearly stated in the manuscript and flowchart.  **Grey literature sources:** Google Scholar, institutional repositories, **Conference abstracts:** screened when methodological detail available, **Manual reference checking:** performed for all included studies  The search was revised in July 2025 to ensure completeness. This update **changed the total number of retrieved records,** but **did not change the number of included studies (n = 14).** | Line 113-116  Line 130-132  Fig1.tiff  Line 163-170 |
|  | Results  The Results section is detailed, thorough, and well-organized, providing granular data on study characteristics, intervention parameters, and outcome domains. However, **it reads more like a systematic synthesis** than a scoping overview.  The authors provide statistical detail and comparative analysis rather than **mapping the breadth of evidence or identifying knowledge gaps,** which limits the alignment with scoping review principles. | We thank the reviewer for this insightful comment. In response, we have **substantially revised the Results section** to ensure it aligns with scoping review methodology and the objectives of evidence mapping.  **Key revisions implemented:**   1. **All inferential or comparative language has been removed.** The revised Results no longer report statistical significance, group comparisons, or performance-related interpretations. 2. **The Results now follow an evidence-mapping format.** Outcomes are described in terms of:    - what was measured,    - how it was measured, and    - where studies reported changes or no changes, without drawing conclusions about effectiveness. 3. **The section has been restructured to emphasise breadth rather than evaluation.** For each ICF domain, we describe:    - the types of measures used,    - the number of studies reporting each outcome,    - the variability in reporting across studies. 4. **Any language implying superiority, benefit, or directionality has been removed.** Terms such as “significant improvement,” “favouring,” or “consistently positive” have been eliminated. 5. **Variability and diversity of measures are highlighted neutrally, reflecting gaps in standardisation.** This aligns with the intention of a scoping review to map—not synthesise—evidence. | Line 251-292 |
|  | Discussion  The Discussion is comprehensive and clinically grounded, effectively situating findings within the broader literature. It highlights heterogeneity in CT delivery and the need for standardised exercise prescriptions.  However, **it again adopts language and interpretation typical of a systematic review, focusing on intervention effectiveness rather than evidence mapping.** The discussion could be improved by **synthesising how study design, CR phase, and delivery setting influence outcomes** and **by restructuring the “gaps” section to align with the PCC (Population, Concept, Context) framework.** | Thank you for this helpful feedback. We have revised the Discussion to adopt a clearer evidence-mapping approach and removed language suggestive of intervention effectiveness. The revised section now synthesises how study design, CR phase, and delivery setting relate to reported outcomes, and the “gaps” subsection has been restructured to explicitly follow the PCC (Population, Concept, Context) framework as recommended. | Line 307-419 |

**RESPONSE TO REVIEWER 2**

| Reviewer #2: Summary | **Comments** | **Rebuttal** | **Location (line numbers)** |
| --- | --- | --- | --- |
|  | This scoping review sought to describe the effects of concurrent training (CT), a combination of aerobic and resistance exercises, across various health-related outcomes compared with standard care in patients diagnosed with coronary artery disease (CAD). The systematic search identified fourteen studies involving a total of 1,126 participants. Overall, the findings suggest that CT leads to improvements in exercise capacity, muscle strength, and functional performance among individuals with CAD. However, its influence on physical activity levels and health-related quality of life remains inconsistent. The authors attributed these mixed results to variations in exercise prescription across studies and highlight the need for more standardised research to clarify the long-term impact of CT in cardiac rehabilitation settings.  This scoping review offers a valuable synthesis of existing literature and identifies key gaps in current understanding of CT’s role in clinical populations. In particular, it provides novel insights by emphasizing the limited evidence surrounding the benefits of CT programs for patients with CAD. Nonetheless, several minor issues and clarifications remain to be addressed to enable a more accurate understanding of the findings. |  | NA |
|  | Comments:  Line 35: Please add a **dot above the “V” in the VO2 abbreviation and ensure that the “2” is consistently formatted as a subscript** throughout the manuscript (e.g., in Table 3). | Thank you for highlighting this formatting issue. We have carefully revised the manuscript to ensure that all oxygen-consumption terminology now follow standard physiological notation. The abbreviation has been corrected to **V̇O₂** throughout the text, tables (including Table 3), and figure captions. | Throughout the manuscript |
|  | Lines 35-36: Could you please clarify **what is meant by moderate and high-intensity format? Does the term “intensity” refer to the aerobic training, the strength training, or both?** | Thank you for the comment. We have revised the abstract to remove the previous phrasing referring to “moderate- and high-intensity formats.” The updated abstract now specifies that the term “intensity” refers to the aerobic-training component of CT, which was the most consistently reported parameter across studies, and describes the variability in aerobic-training intensity using the appropriate physiological anchors (e.g., %V̇O₂peak, %HRmax, %HRR). Strength-training intensity is now reported separately to avoid ambiguity. | Line 34-41 |
|  | Line 48: Change to **“cardiovascular diseases remain a leading…”** (remove the). | We have revised Line 48 accordingly, changing the phrase to **“cardiovascular diseases remain a leading…”** and removed the definite article as recommended. | Line 53-54 |
|  | Lines 61: Consider adding a **definition of concurrent training** here, along with the abbreviation “CT.” | Thank you for the suggestion. We have now added a definition of concurrent training and introduced the abbreviation “CT” in the Introduction. Specifically, we define CT as a programme that incorporates both aerobic and resistance exercises, either within the same session or across the rehabilitation period, rather than aerobic exercise alone, and we use the abbreviation “CT” consistently thereafter. | Line 73-76 |
|  | Lines 109-110: Please **clarify whether four or five databases were used,** as only four examples are cited. Adjust accordingly. | Thank you for pointing this out. We confirm that **four databases** were used in the search strategy. The text has been revised for accuracy and consistency | Line 120 |
|  | Lines 110-111: Could you **clarify what was done in July 2025**? **What revisions were made since October 2023**? | Thank you for noting the ambiguity. We have clarified the search timeline in the Methods section. The main search was conducted from October 2023 to March 2025, followed by an update in July 2025 to capture newly published or newly indexed studies prior to manuscript finalisation. | Line 122-124 |
|  | Lines 111-112: Please **add the range of publication years for the oldest and most recent studies included**. | Thank you for the suggestion. We have added the publication year range for the included studies in the Results section. The revised text now states that the 14 included studies were published between **2007 and 2022.** | Line 169-170 |
|  | Lines 113-114: Add **quotation marks** around “CAD” and “rehabilitation.” | We have added quotation marks around **“CAD”** and **“rehabilitation”.** | Line 127-128 |
|  | Line 116: A **closing bracket** is missing after “fitness.” | We have added bracket after “fitness”**.** | Line 130 |
|  | Line 156: In the abstract, it is mentioned that there were no **adverse events.** However, this is not addressed in the results section. Please clarify. | Thank you for noting this omission. We have now added a dedicated **“Adverse Events”** subsection in the Results section to report safety findings. This section states that none of the included studies reported any intervention-related adverse events, consistent with the statement in the abstract. | Line 293-296 |
|  | Lines 161: Add the **total sample size and the number of female participants**. Additionally, were there **studies that included only male or only female participants**? | Thank you for this helpful suggestion. We have revised the Study Characteristics section to report the total sample size and gender distribution. The text now states that the 14 studies included **1,037 participants in total, comprising 626 males and 309 females.** We also clarify that **10 studies included both genders, 3 studies enrolled only male participants, and 1 study enrolled only female participants.** | Line 178-183 |
|  | Line 177: Could you specify **what is meant by “conducted in separate sessions**”? | Thank you for the comment. We have revised the sentence to improve clarity and to specify what is meant by “conducted in separate sessions.” The updated text now explains that, in this study, aerobic and strength training were performed on different days, with two weekly sessions dedicated to aerobic exercise and two sessions dedicated to circuit-based resistance training | Line 198-200 |
|  | Line 218: This paragraph mentions that **one study did not report a significant improvement in VO2peak compared to aerobic training. What about the other studies**? | Thank you for the comment. We have revised the paragraph to clarify the findings across all studies reporting V̇O₂peak. The updated text now distinguishes studies showing improvement from those reporting no additional benefit when CT was compared with aerobic training, thereby providing a clearer summary of the full range of results. | Line 253-260 |
|  | Lines 310-312: The exercise prescription could also have influenced the results. The mixed findings open the door for future research exploring different combinations of aerobic and strength training. **Does intensity or frequency matter?** | Thank you for the insightful comment. We have revised the paragraph to acknowledge that differences in exercise-prescription characteristics—such as the combination of aerobic and strength modalities, as well as variations in training intensity and frequency—may have contributed to the mixed findings. The updated text also highlights the need for future research to explore how these specific prescription variables influence outcomes. | Line 387-398 |
|  | Line 330: I also think it is important to **highlight the lack of studies examining sex differences in response to combined training (CT).** **Does sex influence the physiological or functional adaptations to CT?** This is an important question that has not been sufficiently addressed or emphasized in this scoping review and warrants further discussion. | We thank the reviewer for this important comment. We have now expanded the Discussion to explicitly address the lack of studies examining sex differences in response to concurrent training. A new paragraph highlights the under-representation of females in existing trials, summarises known differences in baseline fitness and CR outcomes between males and females, and emphasises the need for future research to determine whether physiological and functional adaptations to CT differ by sex. | Line 374-386 |
